# Clinical Reports

**Published:** 1900-02

**Authors:** S. J. J. Harger

**Affiliations:** Veterinary Department, University of Pennsylvania, Philadelphia, Pa.


### CLINICAL REPORTS.

By S. J. J. Harger, V.M.D.,
VETERINARY DEPARTMENT, UNIVERSITY OF PENNSYLVANIA, PHILADELPHIA, PA.

Case I.—Animal, b. g., aged, which I was called to see with Dr. W. H. Mattson, of Chester Heights, Pa.

*History.* The animal last summer suffered from a mild form of strangles, and seemed to recover. Subsequently was noticed to have difficulty in swallowing; slight discharge from the nose continued, which at times became very profuse, as if from pharyngeal abscess; on one occasion there was some hemorrhage from the nose. The difficulty in swallowing was progressive.

*Condition when seen.* Extreme emaciation, temperature 102° F., and in the meantime had picked up a nail. Profuse nasal discharge mixed with food, appetite ravenous, but could swallow only a minimum quantity of food and water; regurgitation of food and water through the nostrils; gradual starvation. Subacute traumatic pneumonia of posterior lobe of left lung.

*Local conditions.* Absence of pain around the throat. The muscular lesions were interesting: the left sterno-maxillaris muscle had almost entirely disappeared, from atrophy. On palpation the left side of the pharyngeal region appeared more hollow than the right; to the fingers the surface and the superior border of the thyroid cartilage of the larynx appeared almost as if this cartilage was subcutaneous, and could be distinctly outlined, showing that the middle and inferior constrictor muscles of the pharynx (thyro-pharyngeus and crico-pharyngeus) were very thin, while on the right side the muscular covering of this cartilage was of normal thickness. Otherwise no external alteration.

The muscular lesion of the pharynx and of the sterno-maxillaris muscle and the intimate relation of the nerves supplying these muscles—the pneumogastric and spinal accessory, both within the cranial cavity and at the base of the skull—led us to conclude that these nerves were implicated, probably by a deep-seated abscess at the base of the brain or in the guttural lymphatic glands.

The nature of the muscular alterations being recognized, and also, considering the duration of the disease, an unfavorable prognosis was unhesitatingly given; the animal was destroyed.

*Post-mortem.* Made by lamplight under many disadvantages.

The diagnosis of the muscular lesions was verified. All the muscles on the left side of the pharynx, as well as of the larynx, had almost entirely disappeared from atrophy. No lesions along the course of the nerves mentioned, or of any other structure beside the muscles named, could be found; but, judging from the distribution of the nerves and the diseased process in the muscles which they supply, there evidently must have been a central lesion. A fact in corroboration of central pressure on the vagus or the medulla was the slowness and *exceeding shallowness* of the respirations, although the lungs were diseased. Unfortunately, the brain could not then be removed, but we know that abscesses complicating strangles may be found in very unusual places, and it is a question whether there may not have been an intracranial abscess involving the medulla. Such may have been possible, since morbid growths developing slowly may exist in the brain for a long time without producing aggravated symptoms. Inquiry as to brain symptoms elicited nothing excepting that the animal on one occasion had a "dizzy spell," and the owner had some difficulty in returning him to the stable.

Masticated food was found in the larynx, which also caused traumatic pneumonia.

CASE II. SCIATIC (FEMORO-POPLITEAL) NEURECTOMY. Animal, sorrel gelding; lame in the near hind member; had been in this condition when purchased, five weeks ago. Local alterations: Periarthritis of the fetlock articulation, involving all its ligaments; periostitis of the lower end of the cannon-bone and the first and second phalanges; legs much enlarged from the hoof to the middle of the cannon. The lameness was very decided, continuous, and when standing the animal would continually hold up the foot. The inflammatory symptoms were of a chronic type. The parts had been blistered without improvement.

The value of the animal and the time required militated against the use of the actual cautery, and resection of the sciatic nerve above the point of the back was decided upon. The operation need not be described here, but, it may be repeated, can be successfully employed in cases in which firing is not an economic procedure. Experience has shown that in such cases resection of the sciatic nerve is a satisfactory method of treatment in such cases, and after this operation horses have for a long time done work of various kinds. After the operation the animal went practically sound.

CASE III. (Same operation as Case II.) Animal, b. g., had been lame since last spring and treated with rest and blistering, and was unable to do any work.

*Symptoms.* Extreme lameness; thickening and induration in the off hind leg of the perforatus and the perforans and its check ligament, which were much contracted. Walking on the toe, unable to place the heel on the ground to within four inches, and the wall at the toe was inclined upward and forward in walking—an aggravated condition identical with that so frequently seen in mules.

Tenotomy was considered too expensive and requiring too long a time to recover. Sciatic neurectomy was decided upon, and a toe-box shoe with high heels applied. The heels immediately afterward were lowered nearer to the ground. After three weeks an ordinary shoe was applied, the heels gradually touched the ground, and at the end of about five weeks he walked and trotted normally, and was able to work. The merest trifle of lameness remained, which there is fair reason to believe will ultimately disappear.

We frequently see cases to which such treatment may be applicable.

CASE IV. RESECTION OF THE SPINAL ACCESSORY NERVE FOR CRIBBING AND ROARING. In communicating with Dr. H. D. Fennimore, of Knoxville, Tenn., on the subject of cribbing I have come into possession of a few very interesting facts which demonstrate that the same operative means that cured the animal from cribbing also entirely relieved him from roaring. I shall not appropriate the doctor's work to my own personal advantage, but knowing that it would be of interest to veterinarians, and fearing that it might remain unpublished, and perhaps may lead to more important results, I shall apologize to him for summarizing as follows:

Animal, b. g., mark of 2.22, ten years old. The animal was an inveterate cribber as well as a decided roarer. The spinal accessory nerve on both sides was resected in the usual manner, with a view of relieving the cribbing, without taking into consideration the respiratory trouble. The result was that the operation not only cured the horse from cribbing, but also from roaring, and "made a very valuable horse of him."

A second case was a valuable bay gelding. A diagnosis of roaring was not thoroughly established, but the symptoms, as far as I can conclude, pointed toward that trouble. The day following that of the operation the horse was taken for a severe drive, and was found to be very much improved. Since that day I have no data as to the result, which, of course, is incomplete.

Such a result it is difficult to interpret anatomically and pathologically. There is no direct relation between the spinal accessory nerve and the lesions of roaring. The only possible effect which the sterno-maxillaris muscle can have upon obstructing the respirations may consist in excessive flexion of the head upon the neck in "drawing in the chin," but even this circumstance is not sufficient to produce symptoms of confirmed roaring, and consequently paralysis of this muscle from resection of its motor nerve-supply would not remove this obstruction to the respiration. To say the least, this is a remarkable coincidence. However, the first opportunity should be seized to experiment with such a simple means of treatment.

Case V. Spurious Rabies. The animal was an English setter dog, with a history that four days before he had been washed with Good's dog soap, which had poisoned him. When first seen he presented all the symptoms usually observed in dumb rabies. He showed that peculiar, miserable-looking expression of the eyes, dropping of the lower jaw, and salivation; at times he would gnaw at the chain to which he was tied; extreme general weakness was marked, and he supported himself against a fence to which he was tied.

In view of suspected rabies, no treatment was prescribed, and a natural termination was awaited. The animal gradually improved, and in four days was well.

The nature of the disease evidently is obscure. That the soap has been the cause is doubtful; it had been repeatedly used on this dog before. The rapid recovery precludes rabies. This is the first case among a great many presenting this train of symptoms that has recovered, and teaches us not to be too hasty in expressing a radical prognosis in such cases without experimental means of confirming the diagnosis, although in similar cases the termination has invariably been fatal. Again, relative to rabies, I have in such cases received reliable histories that precluded any reasonable chance of contact with other dogs; but, nevertheless, experimental inoculation of rabbits confirmed the diagnosis, and absence of association with other dogs should not deserve too much credence. Perhaps we still have to learn unknown conditions of the modes of infection with rabies.

